# Metabolomics Approaches for the Comprehensive Evaluation of Fermented Foods: A Review

**DOI:** 10.3390/foods10102294

**Published:** 2021-09-28

**Authors:** Yaxin Gao, Lizhen Hou, Jie Gao, Danfeng Li, Zhiliang Tian, Bei Fan, Fengzhong Wang, Shuying Li

**Affiliations:** 1 Institute of Food Science and Technology, Chinese Academy of Agricultural Sciences, No. 2 Yuan Ming Yuan West Road, Beijing 100193, China; gaoyx2021@sina.com (Y.G.); houlizhen2021@sina.com (L.H.); gj1781358244@sina.com (J.G.); 82101205002@caas.cn (D.L.); 82101202159@caas.cn (Z.T.); fanbei@caas.cn (B.F.); 2 Key Laboratory of Agro-Products Quality and Safety Control in Storage and Transport Process, Ministry of Agriculture and Rural Affairs, Chinese Academy of Agricultural Sciences, Beijing 100193, China; 3 Key Laboratory of Agro-Products Processing, Ministry of Agriculture and Rural Affairs, Chinese Academy of Agricultural Sciences, Beijing 100193, China

**Keywords:** fermented foods, metabolomics, metabolomic approach, qualitative property

## Abstract

Fermentation is an important process that can provide new flavors and nutritional and functional foods, to deal with changing consumer preferences. Fermented foods have complex chemical components that can modulate unique qualitative properties. Consequently, monitoring the small molecular metabolites in fermented food is critical to clarify its qualitative properties and help deliver personalized nutrition. In recent years, the application of metabolomics to nutrition research of fermented foods has expanded. In this review, we examine the application of metabolomics technologies in food, with a primary focus on the different analytical approaches suitable for food metabolomics and discuss the advantages and disadvantages of these approaches. In addition, we summarize emerging studies applying metabolomics in the comprehensive analysis of the flavor, nutrition, function, and safety of fermented foods, as well as emphasize the applicability of metabolomics in characterizing the qualitative properties of fermented foods.

## 1. Introduction

Fermented foods have been produced and consumed since around the Neolithic period and have been embedded in dietary cultural norms around the world [1,2]. Fermentation preserves food and provides new flavors and textures from traditional foods that contribute to pleasant gastronomic experiences and health benefits [3,4]. Globally, a wide variety of fermented foods commonly present in daily diets, which include those derived from beans, milk, cereals, vegetables, fruits, meat, and seafood [5]. Some commonly consumed fermented foods include miso, soy paste, natto, and vinegar from East and Southeast Asia and yogurt, cheese, and kefir from Europe and America [6,7,8]. Other traditionally fermented foods include pickled fruit and vegetables and fermented beverages (e.g., beer, wine, and tea) from cereals and plants [9,10].

Fermentation is a metabolic process wherein microorganisms enzymatically convert substrates in raw materials into new products, which results in the unique final flavor, nutritional, functional, and safety characteristics of fermented foods [5,11]. The microorganisms used in fermented foods mainly include fungi (e.g., *Saccharomyces* spp., *Mucor* spp., and Rhizopus spp.) and bacteria (e.g., *Bacillus subtilis* spp., *Lactobacillus* spp., and *Bifidobacterium* spp.) [12,13]. Fermented foods have been recognized as key ingredients to develop novel functional foods in the food industry. Fermentation can foster the improvement of food properties, including the transformation of the nutrient profile and the formation of novel flavor, as well as functional and health properties, because microorganisms can generate enzymes that degrade macromolecular compounds and nonnutritive factors (e.g., tannin, protease inhibitor, and soybean agglutinin) in raw materials, to improve the digestion and absorption characteristics [10,14,15,16,17]. In addition, food safety and shelf life can be enhanced by preventing growth of pathogenic microorganisms [18]. Therefore, the actions of microorganisms on the primary and secondary metabolites generated during the fermentation process modify the properties of fermented foods. Recently, researchers have preferred using metabolomics analysis to provide detailed information regarding metabolites (amino acids, lipids, and phytochemicals) and thus effectively evaluate food properties.

Currently, metabolomics is an emerging field but is also considered an established but evolving analytical technology widely involved in many fields such as food science, food quality, and food safety [19,20]. With the continuous advancement of separation techniques and high-resolution mass spectrometry, and improvements in database and data processing software, metabolomics is gradually gaining acceptance as a method to empirically delineate the properties of fermented foods and probe functional metabolomes [21,22]. The concept of metabolomics was first proposed by Nicholson in 1999, as a component of systems biology. It extends the principles of genomics and transcriptomics, enabling qualitative and quantitative analysis of small molecular metabolites (<1500 Da) in biological systems [23,24].

Metabolomics allows for the collection of all metabolites in cells, tissues, or organisms under specific physiological periods or conditions [25]. Metabolomic signatures (fingerprints) can be obtained using different approaches, such as nuclear magnetic resonance (NMR) spectroscopy, gas chromatography–mass spectrometry (GC-MS), liquid chromatography–mass spectrometry (LC-MS), and capillary electrophoresis–mass spectrometry (CE-MS) [26,27,28]. MS-based metabolomics is divided into two types based on whether they are targeted. Non-targeted metabolomics is biased toward high-resolving power, detecting as many metabolites as possible without quantification; involves detectors including time-of-flight (TOF), Orbitrap, and Fourier-transform ion cyclotron resonance (FT-ICR); can identify several thousand metabolites. In contrast, targeted metabolomics aims to achieve highly sensitive detection and quantification, usually targeting and quantifying a selected group of compounds with a triple quadrupole (QQQ) detector [19,28]. The sensitivity, resolution, and metabolite coverage vary among these approaches, based on the corresponding analytical instruments, which often complement each other. However, the primary disciplines utilizing the applications of quantitative and qualitative metabolomics include microbiology [29], plant physiology [30], medicine [31], human health [32], and food and nutritional research [33].

In this review, we assess the latest applications of metabolomics in fermented food research. We discuss several metabolomics approaches and outline the perspectives on the current trends toward applying metabolomics to probe the flavor, nutrition, function, and safety properties of fermented foods. Further, this review provides a reference for quality control and identification associated with fermented foods.

## 2. Methodology

Studies describing food metabolomics have been published in many journals in the same field. Multiple databases were used to conduct a comprehensive search on related topics, such as Science Direct, National Center for Biotechnology Information, Web of science, and Google Scholar. The keywords included metabolomics, food, fermentation, detection methods, flavor, nutrition, function, safety, research, and were combined in different ways to effectively search relevant and up-to-date literature.

## 3. Metabolomics Workflow

**Figure 1:**
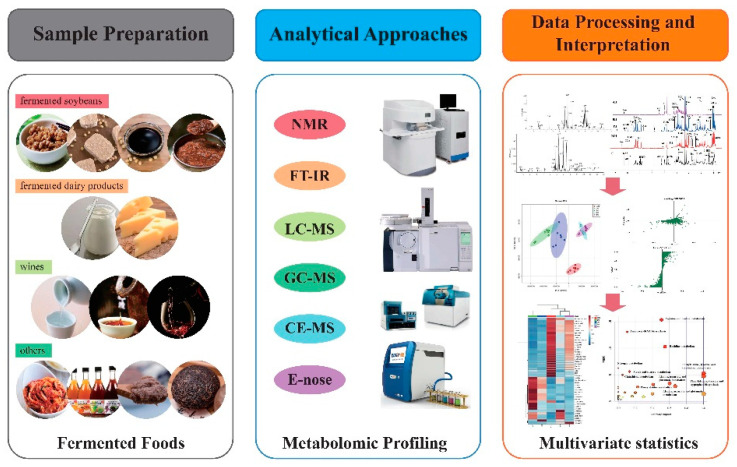
Schematic overview of the most important platforms used for metabolomics studies.

The basic metabolomics workflow was gradually standardized during application development (Table 1). It comprises three key steps: sample preparation, analytical approach, and data processing and biological interpretation (Figure 1, from http://www.google.com, accessed on 30 June 2021) [23,34]. Effective sample preparation is a critical step for subsequent analysis [35]. The methods for collecting and processing samples vary based on the samples or research questions, and mainly involve time-dependent sampling to reflect the temporal dynamics and microbial successions involved in fermentation [36]. Metabolite separation and detection (e.g., NMR and GC-MS) have been recognized as key steps in metabolic profiling [37]. Metabolomics studies, distinguished by different separation and detection methods, have been widely used in foods. They can effectively discriminate and predict potential metabolites and explain the biological processes studied, thus reflecting the food properties, such as flavor, nutrition.

### 3.1. Metabolomic Approaches Based on Different Separations and Instrumentation

#### 3.1.1. NMR-Based Metabolomics

NMR is one of the most frequently used analytical tools in metabolomic fingerprinting and profiling [38]. Several recent publications have described NMR-based metabolomics in plants, animal tissues, foods, and beverages [39]. NMR, which relies on bulky and expensive instruments (magnets and electronics), can interpret biomolecular dynamics with stereochemical detail, and detect all compounds with abundant protons, such as sugars, polyphenols, amino acids, vitamins, nucleotides, and other organic compounds [40,41,42]. Beyond the initial installation costs, the running costs are relatively inexpensive [43]. Moreover, the non-destructive characteristics of the technology have ensured wide adoption among current metabolomic approaches, since sample preparation is relatively simple and does not involve chemical extractions, which contributes to the ability to analyze a single sample in multiple consecutive experiments [44,45]. Moreover, with minimal sample preparation, NMR technology enables simultaneous identification and absolute quantification of compounds in a sample based on the direct relationship between molar concentration and the intensity of the NMR resonances, achieving high-throughput detection [46]. However, the lower sensitivity and large sample quantity requirements are the principal drawbacks of NMR [47,48]. In addition, high-resolution NMR spectroscopy (HRMAS-NMR) has become a rapid and accurate alternative technique at the molecular level, following the development of high-field NMR spectrometers [49]. In recent years, NMR spectroscopy has been widely applied to analyze the metabolomes of traditional foods such as soy sauce [50], fermented fish sauce [51], and alcoholic beverages [49].

#### 3.1.2. FT-IR-Based Metabolomics

Infrared (IR) spectroscopy is considered an effective tool for classifying, characterizing, and identifying small molecules, and FT-IR spectroscopy has rapidly become a valuable metabolic fingerprinting tool [52,53,54]. FT-IR enables the simultaneous and accurate measurement of major and minor components, such as carbohydrates, polysaccharides, polyphenols, proteins, amino acids, and fatty acids [55,56,57]. Similar to NMR spectroscopy, FT-IR spectroscopy can also be performed in a rapid and non-destructive manner without sample extraction and derivation on either liquid or solid samples [58]. It is often applied for rapid detection of adulteration and evaluation of the maturity of foods (e.g., sesame oil, mango) [59,60,61]. However, its major disadvantage is the inability to distinguish the chemical constituents of a mixture [62]. Thus, FT-IR is generally combined with other analytical techniques to provide more accurate information on elemental composition and molecular structure, resulting in the identification of the main metabolites (e.g., sugars and organic acids) found in fermented foods [54,63,64].

#### 3.1.3. GC-MS/LC-MS-Based Metabolomics

Over the past decades, MS-based chromatographic systems (GC or LC) have rapidly developed into the most commonly used analytical approaches in metabolomic studies, partly because they yield higher sensitivity than the previously discussed techniques [35,65,66]. The crucial component for GC-MS and LC-MS operation is the mass detector, including QQQ, TOF, and electrospray-ionization (ESI), each of which have advantages depending on the specific applications and sample types [67,68]. The choice of chromatographic separation for metabolite analysis is related to the physicochemical properties of the compounds, and both approaches have their own related advantages and disadvantages.

GC-MS is the preferred analytical technique for the study of volatile compounds and is mainly employed for evaluating primary metabolites such as amino acids, fatty acids, organic acids, alcohols, and sugars or sugar alcohols [45,69]. GC-MS-based metabolomics provides high sensitivity and highly repeatable fragmentation, and takes advantage of well-established spectral databases, such as the National Institute of Standards and Technology (NIST), Wiley, and the Automated Mass spectral Deconvolution and Identification System (AMDIS), allowing for the identification of important biomarkers [70]. However, one drawback of GC-MS-based metabolomics is the complex pre-treatments required before analysis, followed by difficult sample processing and derivatization required to modify polarity and facilitate effective separation [71,72]. Thus, the long detection time required for this approach may not meet the requirements for the rapid detection of many analytes. In contrast, LC-MS is the most widely used technique in metabolomics for profiling soluble and/or unknown metabolites and is commonly used for evaluating secondary metabolites such as flavonoids, saponins, alkaloids, phospholipids, and polyamines [45]. Similar to GC-MS, LC-MS-based metabolomics is also characterized by high sensitivity, robustness, and versatility. Notably, volatility is not required in this approach, allowing for more extensive metabolite detection. The sample derivatization process can be omitted, reducing background signal noise and providing higher quality data [73,74]. Owing to higher separation and sensitivity, GC-MS/LC-MS-based metabolomics has been widely applied to study various complex food matrices, such as dry-cured hams [75], tea [76], honey [77], as well as fermented foods [78,79].

#### 3.1.4. CE-MS-Based Metabolomics

CE-MS, another approach in metabolomics, is now a widely used analytical separation technique, notable for the separation of highly polar and charged metabolites, based on varying mass-to-charge ratios, such as amino acids, organic acids, and nucleotides [80,81]. The most attractive aspect of CE-MS is that the proposed workflow only requires small amounts of organic reagents. This facilitates convenient analysis of volume-limited biological samples, providing rapid yet comprehensive profiling of compounds from small inorganic ions to large proteins [82]. However, the low sample throughput in CE-MS results in relatively poor concentration sensitivity, leading to lower detection sensitivity and reproducibility. In this sense, compared with other analytical separation techniques, metabolome profiling studies using CE-MS lack representativeness, which limits its application [83]. In contrast with GC-MS and LC-MS approaches that require interaction with a stationary phase, separation by CE relies on a small electrolyte-filled capillary. Most metabolites are too polar or ionic to be retained by the columns used in GC- and LC-MS [37]. Often, samples that cannot be separated by GC- or LC-MS can be easily resolved by CE-MS [84]. Thus, CE-MS with higher separation is considered a complementary tool to traditional chromatographic techniques, thus improving the efficiency and abundance in the metabolite detection spectra [21]. Over the past few years, recent developments in CE-MS instrumentation for sample throughput and quality control have been achieved. The usefulness of CE-MS as an independent or supplementary technique is gradually gaining traction in the fields of biomedical, clinical, microbial, plant, and food metabolomics [85].

#### 3.1.5. Electronic Nose-Based Metabolomics

Another important detector with applications in metabolomics research is the electronic nose (E-nose), which deploys electronic sensors to mimic the biological olfactory mechanism to obtain the overall odor of products [86,87]. The common E-nose comprises an aroma extraction system, sensor array, analog-to-digital system, and a method for pattern recognition, allowing it to recognize simple or complex, natural or synthetic organic odor compounds but not specific metabolites [88,89]. In this context, the volatile compounds detected by an E-nose exhibit a unique composition, such that they can be used for quality change detection and identification. The E-nose approach has been extensively used in many industries, such as grain [90], dairy [91], and pharmaceuticals [92]. In comparison with traditional high-throughput metabolomic techniques, E-nose technology is easy to operate, offers a lower cost alternative, and non-destructive detection; however, E-nose-based metabolomics technology is still considered to be in its infancy [93]. Furthermore, the use of E-nose technology can quickly achieve real-time monitoring of the quality of agricultural products compared to GC-MS [94]. Combining E-nose technology with MS has been successfully used to distinguish the quality, aroma, and origin of fermented foods, including apple cider vinegar and soybean paste [95,96].

### 3.2. Metabolomic Analyses Based on Data Interpretation and Multivariate Statistics

Metabolomics generates extensive datasets that require effective data processing methods to successively interpret their biological implications. Bioinformatics and multifunctional tools for performing compound identification, statistics, enrichment, pathway prediction, and visualization provide high-quality and comprehensive metabolomics information (Table 1).

Before pattern recognition analysis (multivariate statistical analysis), raw metabolomics data must be pre-processed, and a series of adjustments such as baseline correction, peak recognition, peak alignment, and normalization must be performed using the appropriate software, such as MestreNova (Mestrelab Research, Santiago de Compostela, Spain) and Progenesis QI (Waters Corp., Milford, MA, USA) [97,98]. Subsequently, metabolite identification and characterization are completed based on accurate mass, secondary fragments, and isotope distribution, through gradually standardized online software or websites (e.g., the Human Metabolome Database, HMDB, https://www.hmdb.ca, accessed on 30 June 2021; METLIN Database, https://metlin.scripps.edu/, accessed on 30 June 2021) [98]. The software used may have a great influence on data processing and results; it is important to select an appropriate software package according to different metabolomics methods and research objectives.

In addition, the application of multivariate statistical analysis can effectively reduce the complexity of a reliable metabolomics dataset containing qualitative and quantitative information in interpreting complex biological systems or related biological processes [99,100]. To date, the multivariate statistical tools developed, mainly through SIMCA-P software (Umetrics, Sweden), comprise three major techniques: unsupervised, supervised, and pathway analysis [101]. Principal component analysis (PCA), the most commonly used unsupervised tool, converts a large number of related datasets into a limited number of variables to visualize the most important trends after dimensionality reduction [102]. Partial least squares discriminant analysis (PLS-DA) and orthogonal partial least squares discriminant analysis (OPLS-DA) are usually used to assess obvious differences between the groups (variables) within datasets and identify the biomarkers through the value of variables of importance in projection (VIP) [102,103]. Regarding pathway analysis, pathway enrichment analysis of differential metabolites is mainly based on the Kyoto Encyclopedia of Genes and Genomes (KEGG, https://www.genome.jp/kegg/, accessed on 30 June 2021) database or MetaboAnalyst (https://www.metaboanalyst.ca/, accessed on 30 June 2021), which helps understand the mechanisms of metabolic pathway changes in a sample [99]. Therefore, a reasonable and effective data processing method for metabolomics can maximize the interpretation of the biological significance of the sample and predict the properties of fermented foods [104,105].

**Table 1:** Computational tools for metabolomics analysis.

Biobanks	Role	References
MestreNova	Data processing prediction, publication, verification; data storage and retrieval	[97]
Progenesis QI	Data processing and normalization; qualitative, quantitative and identification of small molecules with significant changes	[98]
SIMCA	Multivariate statistical analysis; pattern recognition of PCA, PLS-DA, OPLS-DA	[97,99,100,101]
RStudio	Multivariate tool; heatmap of metabolites and their concentration changes	[97]
HMDB	Physicochemical and biological properties; biomarker discovery; metabolic pathway information	[97,98,99,100,101]
METLIN	Metabolite identification; metabolite structure; links to other databases	[98,100,101]
PubChem	Biological properties of organic small molecule; metabolite structure; links to other databases	[100]
MetaboAnalyst	Data analysis, visualization, and functional annotation; multivariate analysis; metabolite significance; pathway identification	[98,99,101]
KEGG	Metabolic pathway; metabolite interactions; delivery of gene to metabolite function	[98,99,101]
Mev	Hierarchical cluster analysis; heatmap of metabolites and their concentration changes	[100]
Cytoscape	Interaction network visualization: correlation analysis linked to gene, protein, and metabolite expression	[99]

Note and Abbreviations: SIMCA, soft independent modeling of class analogy; Mev, Multiple Experiment Viewer.

## 4. Applications of Fermented Foods

With increased consumption and a societal increase in health consciousness, the general population is more concerned about whether food and nutrition play an important role in maintaining health and reducing the risk of various diseases. The rise in health awareness has driven tremendous public interest in fermented foods, which have multiple health benefits, including antioxidant, anti-cancer, anti-inflammatory, and cardioprotective properties [106,107]. Fermentation can influence the improvement of organoleptic characteristics, nutritional and functional properties, and safety considerations with a final positive effect on human health, mainly because of the secondary metabolites generated from the interaction of raw materials and probiotics [108,109]. Metabolomic profiling of fermented foods has been used to monitor metabolites representative of qualitative properties during fermentation to predict nutritional, functional, flavor, and safety characteristics of the final products [109,110].

### 4.1. Flavor

The flavor of fermented foods is mostly generated through microbial metabolism and enzymatic biochemical reactions [111]. It is a key indicator of sensory qualities, which often directly determines acceptance by consumers [112]. Characterization of fermented food flavor also contributes to determining the maturity of a product and the differences among products obtained from different production locations or fermentation strains [112,113]. It mainly depends on the composition of volatile flavor compounds, which constitute a preferred flavor, or an unpleasant flavor based on different composition conditions. Flavor components are usually identified by GC-MS [114,115]. The most relevant information obtained from metabolomic studies on the flavors of fermented foods is presented in Table 2.

Distinctive flavors are essential factors in alcoholic beverages, including *baijiu*, *huangjiu*, beer, and red wine, which are all commonly consumed in Chinese society. Yang et al. [116] applied GC-GC-TOF-MS to analyze *baijiu* made from yellow, white, and black daqu, and captured 401 volatile compounds, mainly aromatic compounds and pyrazines. Among them, white daqu has the most types of esters and alcohols, but the levels of esters and alcohols in yellow and black daqu were higher than in white daqu, which may be related to microbial metabolism during fermentation. The flavor of Chinese *huangjiu* from Shanxi, a yellow rice wine with zao-aroma, was also analyzed based on GC-MS and GC-olfactometry (GC-O) techniques, and ethyl cinnamate and ethyl 3-phenylpropionate were demonstrated to be key aroma compounds, which indicated differences from others such as Zhejiang Shaoxing and Fujian Hongqu *huangjiu* [112,115]. Pu-erh tea is a unique tea obtained from the fermentation of *Monascus* tea, and the understanding of the formation and evolution mechanism of its flavor-active compounds can improve its quality. Deng et al. [117] summarized the metabolic evolution of key flavor-active compounds during Pu-erh tea fermentation systematically based on headspace solid phase microextraction (HS-SPME)-GC-MS. The volatile fingerprint and flavor profiles of fermented soybean foods have been investigated using metabolomics technologies. The flavor of fermented soy foods significantly reduces the proportion of beany flavor substances in the soybean itself, which is an unpleasant flavor affecting its consumption [118]. Wangzhihe red sufu, a Chinese traditional fermented condiment, was analyzed using metabolomics during the fermentation process, and four typical commercial red sufu products were detected by GC-MS, GC-O/MS, and E-nose [119]. The most prevalent volatile components (e.g., phenolics, esters, and alcohols) varied in the molded phetze, salted phetze, and post-fermentation stages of red sufu, and there were differences in the overall volatile profiles of the four sufu products, which were conducive to effective discrimination of the extent of sufu maturity and the type of sufu products. Notably, the flavor of products related to natto, and soybeans fermented by *B. subtilis*, are usually undesirable to the Chinese population, whereas natto is very popular in Japan where it is an important side dish. The volatile metabolite composition of natto products has been investigated through GC-MS and NMR, and it has been shown that ammonia, 2,5-dimethylpyrazine, isovalerate, isobutyrate, and 2-methylbutyrate are increased in amount and proportion in natto, which may explain the undesirable flavor [120,121,122]. Gao et al. [122] studied soymilk fermented by *B. subtilis* through NMR and not only found undesirable flavor compounds but also explained the changes in nutrients and functional components in fermented soymilk. In addition, metabolomics has played a key role in determining the maturity of cheese and other dairy products [113], the analysis of the correlation between flavor components and microbial communities in the types of fermented mandarin fish [123], and the elucidation of dynamic changes in flavor composition during the fermentation of rose vinegar and shrimp paste [124,125].

**Table 2:** Metabolomics applied to different fermented foods on the flavor property.

Fermented Foods	Microorganisms	Metabolomic Analysis		References
		Techniques	Compounds/Properties Analyzed	
Dajiang	Yeast, *Aspergillus, Mucor, Rhizopus, Lactobacillus, Tetragenococcus*	HS-SPME/GC-MS	Alcohols, esters, phenolic acids, aldehydes, ketones	[78]
Red sufu	*Monascus purpureus, Aspergilus oryzae, Actinomucor elegans*	GC-MS, GC-MS-O, E-nose	Amino acids, organic acids	[119]
Natto	*Bacillus subtilis*	GC-MS; NMR	Amino acids, organic acids, pyrazines; ammonia	[120,121,122]
Cheese	*Lactic acid bacteria*	HS-SPME/GC-MS, FT-IR, E-nose	Lactose, lactate and citrate, amino acids, fatty acids	[113]
Huangjiu	Yeast	GC/GC-MS, GC-O	Esters, linalool, neroidol, geranyl acetone, 2-pentyl-furan, methanethiol	[112,115]
Baijiu	Yeast, *Lactobacillus,* *Acetobacter*	GC×GC-TOF/MS	Aromatic compounds, pyrazines	[116]
Red wine	Yeast	HS-SPME-Arrow-GC-MS/MS	Piperitone, mintlactone, menthyl acetate, neomenthyl acetate	[79]
Vinegar	*Acetobacter, Lactobacillus*	HS-SPME/GC-MS	Ethyl acetate, phenylethyl alcohol, acetoin, acetic acid	[124]
Pu-erh tea	*Monascus purpureus, Bacillus, Rasamsonia, Lichtheimia, Debaryomyces*	HS-SPME/GC-MS	β-damascenone, methoxybenzene, 2,4-nonadienal, terpinene, linalool	[114,117]
Siniperca chuatsi	*Psychrilyobacter, Fusobacterium, Vibrio*	HS-SPME/GC-MS	Alcohols, hydrocarbons, nitrogen compounds	[123]
Shrimp paste	*Salimicrobium, Lentibacillus, Lactobacillus, Tetragenococcus*	HS-SPME/GC-MS	Alcohols, aldehydes, nitrogen compounds	[125]

### 4.2. Nutrition and Function

Consumer interest “beyond nutrition” and “functional” foods has surged dramatically, and fermented foods occupy a significant space in the functional foods market [126,127]. Controlling the growth of natural microorganisms or employing probiotics during fermentation, can improve the nutritional and functional value of food [128]. The functions of fermented foods are mainly related to the biological reactions caused by enzymes produced by microorganisms. On the one hand, the basic nutrients of the substrate (e.g., proteins, carbohydrates, and lipids) are degraded into more basic molecular components (e.g., peptides, amino acids, monosaccharides, and fatty acids) or undergo configuration changes (e.g., glycoside to aglycon). However, specific functional components (e.g., menadione and nattokinase) of microbial metabolism are produced. The metabolites (such as genistein, tea polyphenols, selenium) constitute the dietary components of fermented foods, which will affect the epigenetic mechanisms (such as DNA methylation, histone modification, and demethylation) driven by intestinal microbiota, lymphocytic cells, and cancer cells in vivo, conferring fermented foods with antioxidant, anti-inflammatory, antihypertensive, anti-cancer, and neuroprotective properties [129,130,131], thus extending their use as nutraceuticals. The application of metabolomics to decipher nutritional and functional components has recently increased (Table 3). Currently, attention on fermented functional foods is mainly focused on fermented bean products, fermented dairy, and alcoholic beverages.

Fermented soy products, including miso, meju, soybean paste, tempeh, and natto, are a staple in daily cuisine in East Asian countries. Based on metabolic characterization, it was found that during fermentation, formation and abundance of small peptides, amino acids, and fatty acids through the degradation of macromolecular substances such as protein and fat have an important influence on the nutritional quality of fermented soy foods [132,133]. With the extended fermentation time, the composition ratio of the main metabolites will change, and the level of functional active ingredients (e.g., γ-aminobutyric acid (GABA) and isoflavones) will increase, improving the nutritional value and biological activity, potentially benefiting human health [21,122,134]. Kim et al. [135] applied GC-MS and ultra-performance liquid chromatography (UPLC)-Q-TOF-MS to identify doenjang metabolites; they determined that amino acids and sugars are the main nutrients, while increasing levels of isoflavones and soyasaponins after the configuration changes exerted antioxidant properties and improved digestibility. Subsequently, some researchers attempted metabolic tracking of isoflavones in healthy adults after consumption of fermented soy foods [136]. The study used NMR and UPLC-MS to monitor the changes in isoflavones in the food, plasma, and urine after consumption of fermented or non-fermented soybeans, confirming that the bioavailability of isoflavones in fermented soybeans increased with a positive impact on health. Another study used metabolomics based on ultra-high performance liquid chromatography (UHPLC)-Q-TOF-MS/MS to identify possible antihypertensive compounds in Korean fermented soybeans [131], analyzed changes in the levels of amino acids and lipids, and determined that levels of phenolic compounds, GABA, antioxidants, and antihypertensive peptides after fermentation were high.

Fermented dairy products in the market are mainly yogurt and cheese, both of which are fermented by lactic acid bacteria, such as *Lactobacillus bulgaricus* and *Streptococcus thermophilus* [137,138]. The probiotic effects of fermented foods have been reported; different fermentation strains and conditions affect the nutritional and functional quality of the final products [139]. The ripening process of cheese has been metabolically fingerprinted using NMR [137]. As the ripening period increases, the metabolic profile showed a distinct pattern of change. Carbohydrates, organic acids, amino acids, and derivatives mainly contribute to the nutritional value of cheese. In addition, fermentation temperature has a significant impact on yogurt quality, which affects microbial growth [140]. Metabolomic profiles were evaluated for yogurt at different temperatures based on UPLC-QQQ-TOF-MS. Lipids and lipid-like molecules were the main metabolites, and amino acids and peptides were the second most abundant; both of these were generated by lipid metabolism and protein degradation. These are more conducive to digestion and absorption and confer health benefits by reducing blood pressure and regulating immunity [139,141,142].

Alcoholic beverages are prepared from plant sources such as grapes and cereals, the characteristics are mainly affected by yeast fermentation. Sugars represent the main groups of metabolites in wines, while organic acids, amino acids, and phenolic compounds represent other minor metabolites, which together constitute the basic nutritional quality of wines fermented with yeast strains [143]. Grapes and red wines are the major dietary sources of stilbenes, which influence human health through antioxidant, anti-obesity, anti-tumor, and neuroprotective effects, with resveratrol as a typical representative compound [144,145]. HPLC-MS is the most commonly applied technique for determining phenolic compounds, particularly stilbenes [146]. Tabago et al. [49] successfully summarized the potential of NMR metabolomics in terms of geographical origin, variety, vintage, fermentation, and quality evaluation of wine and alcohol, to distinguish wines under different fermentation conditions such as bacterial and yeast strains and agricultural cultivars [147].

In addition, metabolomics has been applied to cabbage vinegar [148], Chinese fermented fish sauce [23], Chinese Pu-erh tea [105], and pickles [149] to characterize the key chemical components related to nutrition and function, providing a reference for the development of functional foods.

**Table 3:** Metabolomic analysis techniques applied to different fermented foods considering nutritional and functional properties.

Fermented Foods	Microorganisms	Metabolomic Analysis		References
		Techniques	Compounds	
Meju	*Bacillus* sp., *Mucor* sp., *Aspergillus* sp.	UPLC-Q/TOF-MS	Small peptides, amino acids, GABA	[132]
Doenjang	*Penicillium glabrum, Aspergillus oryzae*	GC-TOF-MS, UPLC-Q/TOF-MS	Amino acids, organic acids, sugars and sugar alcohols, isoflavones	[133,134,135]
Cheonggukujang (or miso, natto)	*Bacillus subtilis, Mucor* sp., *Bacillus* sp., *Aspergillus* sp.; *E. faecium*	UPLC-Q/TOF-MS	Phenolic compounds, peptides, GABA	[131,136]
Cheese	Lactic acid bacteria	NMR	Lactose, uridine diphosphate-hexose, amino acids, organic acids,	[137]
Yogurt	Lactic acid bacteria	UPLC-Triple/TOF-MS	Lipids, lipid-like molecules, small peptides, amino acids, GABA	[98,139]
Wines	Yeasts, *Lactobacillus,* *Acetobacter*	NMR, FT-IR; GC/GC-TOF/MS; HPLC-MS;	Polyphenols, amino acids, ethanol, resveratrol, stilbenes	[49,116,146,147]
Cabbage vinegar	*Lactobacillus, Acetobacter*	NMR, GC-MS	Organic acids, alcohols, sulfides (dimethyl sulfide, dimethyl disulfide, and dimethyl trisulfide)	[148]
Pu-erh tea	*Aspergillus pallidofulvus, Aspergillus sesamicola, Penicillium manginii*	UHPLC-Q/TOF-MS	Phenolic compounds, amino acids	[105]
Fermented fish sauce	*Firmicutes, Proteobacteria, Fusobacteria*	UHPLC-Q/TOF-MS	Small peptides, amino acids	[23]
Pickle nozawana	*Latilactobacillus curvatus, Levilactobacillus brevis*	NMR, GC-MS	Organic acids, GABA, choline, 2,3-butanedione, acetoin, ethyl acetate	[149]

### 4.3. Safety

Consumers not only pay attention to the sensory and nutritional value of food but now also have an increasing awareness of food safety, encouraging the food industry to establish and maintain product characteristics [150]. Highly complex biological changes are usually encountered during the fermentation process, and metabolomic analyses by NMR or LC-MS can explain safety properties of fermented products indicating whether fermentation improves the safety or generates toxic compounds. The most relevant studies on the safety of fermented foods based on metabolomic analyses are presented in Table 4.

The improvement in food safety is mainly reflected in the changes in the nonnutritive factors or toxic compounds of the raw materials (e.g., soyasaponins and alkaloids) before and after fermentation, which improve digestion and absorption. Seo et al. [151] performed MS-based metabolomics on koji fermentation with different substrates (soybean, rice, and wheat) and microbial inoculation. The breakdown of glycosides to free compounds, including soyasaponins and flavonoids, is obvious during fermentation, revealing the nutritional, functional, and consumer safety aspects of fermented foods. Kasprowicz-Potocka et al. [152] concluded that the reduction of alkaloids occurs in fermented lupin meal by yeasts. Nevertheless, a few compounds are not conducive to food quality during fermentation. Biogenic amines and ethyl carbamate carry a certain toxicity in fermented foods (e.g., miso soy paste and fermented fish) and have been detected with LC-MS, CE-MS, and GC-MS-based metabolomics [21,23]. However, probiotics influenced the levels of methylamine, putrescine, ethyl paraben, and ethyl carbamate [37,153]. In addition, metabolomics approaches have also been widely used in the detection of chemical preservatives in fermented foods, including shrimp paste, pickles, soy sauce, and fish sauce [154]. Metabolites from pathogenic microorganisms can be rapidly determined using metabolomic approaches, whereas the development is still in the early stages [155].

**Table 4:** Metabolomic analyses applied to different fermented foods considering safety properties.

Fermented Foods	Microorganisms	Metabolomic Analysis		References
		Techniques	Compounds/Components Analyzed	
Doenjang	*Bacillus subtilis, Rhizopus, Mucor, Aspergillus* sp.	GC-MS	Soyasaponins	[134]
Cheonggukjang	*Bacillus* sp.	GC-TOF-MS, CE-TOF-MS	Soyasaponins	[21]
Koji	*Aspergillus oryzae, Bacillus amyloliquefaciens*	GC-TOF-MS, UHPLC-MS/MS	Soyasaponins	[151]
Vinegar	*Bacillus subtilis, Rizhopus, Mucor, Aspergillus* sp.	GC-MS	Benzoic acid, sorbic acid, dehydroacetic acid, ethyl paraben	[153]
Fermented fish sauce	*Firmicutes, Proteobacteria, Fusobacteria*	UHPLC-Q/TOF-MS	Trimethylamine N-oxide, putrescine, cadaverine	[23]
Shrimp paste, Pickles	*Lentibacillus, Lactobacillus, Latilactobacillus, Tetragenococcus*	GC-FID	Benzoic acid, sorbic acid, propionic acid	[154]
Cheese	Lactic acid bacteria	GC/LC-MS, NMR, FI-TR	Pathogenic bacteria and its metabolites	[155]

## 5. Conclusions and Future Prospects

As the population and health awareness continue to increase, there will be an increasing need for functional as well as nutritious, safe, and palatable foods to meet changing consumer preferences. Fermentation technology can become a source of such functional foods, including the industrial production of traditional fermented foods, the development of fermented foods based on new raw materials, and the improvement of existing food fermentation technologies. Currently, fermented foods usually contain one or more probiotics beneficial to health. Fermentation is a highly complex biological process, which is essential for imparting new flavor characteristics to fermented foods, improving the nutritional and functional properties of raw materials, and improving the biological safety of fermented products. The industrial production and promotion of fermented foods is dependent on understanding its qualitative properties, which may require the introduction of new application technologies for characterization.

The characterization should mainly include the main metabolites and secondary metabolites in the fermented foods, which directly reflect the quality characteristics, such as flavor and nutrition. Based on the established analytical techniques and knowledge of systems biology, metabolomics may prove to be an indispensable technique as it allows simultaneous qualitative and quantitative analysis of all low-molecular-weight metabolites in a certain organism or cell during a specific physiological period, it is also a manifestation of protein and gene level results. Thus, it can help consumers have more direct access to products with dietary nutrients that they desire. Thus, the application of metabolomics combined with multivariate statistical analysis has evolved into a useful tool in food science.

This review shows the importance of applying metabolomic analysis to identify the quality characteristics of fermented foods. It was possible to perceive from the latest research in this field that the existing techniques used in metabolomic studies for investigating the molecular fingerprints of fermented foods include NMR, FT-IR, LC-MS, GC-MS, CE-MS, and E-nose. Each technique has its advantages and disadvantages in terms of sensitivity, reliability, and sample preparation; therefore, the use of a combined method is recommended to provide a more comprehensive understanding of the metabolomes of fermented foods. The application of metabolomics in characterizing the qualitative properties of fermented foods, specifically flavor, nutrition, function, and safety was also summarized in this review, and may assist in the discovery of biomarkers that discriminate maturity, variety, region, and promotion of value-added fermented foods. Nevertheless, considering the complexity of fermented foods and metabolomics, the verification of functional biomarker compounds in fermented foods, development of metabolomics technology for rapid and accurate detection, and integration with different techniques will be required in further research. Moreover, the development and improvement of a reliable database of fermented food metabolites is necessary for practical and industrial applications.

## Data Availability

Not applicable.

## References

[1] Bourdichon F., Casaregola S., Farrokh C., Frisvad J.C., Gerds M.L., Hammes W.P., Powell I.B. (2012). Food fermentations: Microorgan-isms with technological beneficial use. Int. J. Food Microbiol..

[2] Anyogu A., Olukorede A., Anumudu C., Onyeaka H., Areo E., Adewale O., Odimba J.N., Nwaiwu O. (2021). Microorganisms and food safety risks associated with indigenous fermented foods from Africa. Food Control..

[3] Marco M.L., Heeney D., Binda S., Cifelli C.J., Cotter P., Foligne B., Gänzle M., Kort R., Pasin G., Pihlanto A. (2017). Health benefits of fermented foods: Microbiota and beyond. Curr. Opin. Biotechnol..

[4] Terefe N.S., Augustin M.A. (2019). Fermentation for tailoring the technological and health related functionality of food products. Crit. Rev. Food Sci. Nutr..

[5] Gille D., Schmid A., Walther B., Vergères G. (2018). Fermented Food and Non-Communicable Chronic Diseases: A Review. Nutrients.

[6] Jayachandran M., Xu B. (2018). An insight into the health benefits of fermented soy products. Food Chem..

[7] Gao X., Zhang J., Liu E., Yang M., Chen S., Hu F., Ma H., Liu Z., Yu X. (2019). Enhancing the taste of raw soy sauce using low intensity ultrasound treatment during moromi fermentation. Food Chem..

[8] Dong Z.Y., Liu Y., Xu M., Zhang T.H., Ren H., Liu W., Li M.Y. (2020). Accelerated aging of grape pomace vinegar by using additives combined with physical methods. J. Food Process. Eng..

[9] Nwaiwu O., Itumoh M. (2017). Chemical Contaminants Associated with Palm Wine from Nigeria Are Potential Food Safety Hazards. Beverages.

[10] Adebo O.A., Njobeh P.B., Adebiyi J.A., Gbashi F., Phoku J.Z., Kayitesi E., Hueda M.C. (2017). Fermented pulse-based food products in developing nations as functional foods and ingredients. Functional Food-Improve Health through Adequate Food.

[11] Campbell-Platt G. (1994). Fermented foods-a world perspective. Food Res Int..

[12] Gupta S., Abu-Ghannam N. (2012). Probiotic Fermentation of Plant Based Products: Possibilities and Opportunities. Crit. Rev. Food Sci. Nutr..

[13] Pereira G., Neto D.P.D.C., Junqueira A.C.D.O., Karp S.G., Letti L.A.J., Júnior A.I.M., Soccol C.R. (2019). A Review of Selection Criteria for Starter Culture Development in the Food Fermentation Industry. Food Rev. Int..

[14] Dorđevi’c T.M., Šiler-Marinković S.S., Dimitrijević-Branković S.I. (2010). Effect of fermentation on antioxidant properties of some cereals and pseudo cereals. Food Chem..

[15] Saharan P., Sadh P.K., Duhan J.S. (2017). Comparative assessment of effect of fermentation on phenolics, flavanoids and free radical scavenging activity of commonly used cereals. Biocatal. Agric. Biotechnol..

[16] Thirunathan P., Manickavasagan A. (2018). Processing methods for reducing alpha-galactosides in pulses. Crit. Rev. Food Sci. Nutr..

[17] Gänzle M. (2020). Food fermentations for improved digestibility of plant foods-an essential ex-situ digestion step in agricultural societies?. Curr. Opin. Food Sci..

[18] Xiang H., Sun-Waterhouse D., Waterhouse G., Cui C., Ruan Z. (2019). Fermentation-enabled wellness foods: A fresh perspective. Food Sci. Hum. Wellness.

[19] González-Peña D., Brennan L. (2019). Recent Advances in the Application of Metabolomics for Nutrition and Health. Annu. Rev. Food Sci. Technol..

[20] Cook P.W., Nightingale K.K. (2018). Use of omics methods for the advancement of food quality and food safety. Anim. Front..

[21] Kim J.Y., Choi J.N., John K.M., Kusano M., Oikawa A., Saito K., Lee C.H. (2012). GC-TOF-MS- and CE-TOF-MS-based metabolic pro-filing of cheonggukjang (fastfermented bean paste) during fermentation and its correlation with metabolic pathways. J. Agr Food Chem..

[22] Lee D.E., Shin G.R., Lee S., Jang E.S., Shin H.W., Moon B.S., Lee C.H. (2016). Metabolomics reveal that amino acids are the main con-tributors to antioxidant activity in wheat and rice gochujangs (korean fermented red pepper paste). Food Res. Int..

[23] Wang Y., Li C., Li L., Yang X., Chen S., Wu Y., Zhao Y., Wang J., Wei Y., Yang D. (2019). Application of UHPLC-Q/TOF-MS-based metabolomics in the evaluation of metabolites and taste quality of Chinese fish sauce (Yu-lu) during fermentation. Food Chem..

[24] He S., Wang Y., Xie J., Gao H., Li X., Huang Z. (2020). 1H NMR-based metabolomic study of the effects of flavonoids on citrinin production by Monascus. Food Res. Int..

[25] Zhao G., Zhao W., Han L., Ding J., Chang Y. (2020). Metabolomics analysis of sea cucumber (*Apostichopus japonicus*) in different geo-graphical origins using UPLC-Q-TOF/MS. Food Chem..

[26] Fuhrer T., Zamboni N. (2015). High-throughput discovery metabolomics. Curr. Opin. Biotechnol..

[27] González-Peña D., Dudzik D., Colina-Coca C., Ancos B.D., García A., Barbas C., Sánchez-Moreno C. (2015). Evaluation of onion as a functional ingredient in the prevention of metabolic impairments associated to diet-induced hypercholesterolaemia using a multiplat-form approach based on LC-MS, CE-MS and GC-MS. J. Funct Foods.

[28] Pezzatti J., Boccard J., Codesido S., Gagnebin Y., Rudaz S. (2020). Implementation of liquid chromatography-high resolution mass spec-trometry methods for untargeted metabolomic analyses of biological samples: A tutorial. Anal. Chim Acta..

[29] Mapelli V., Olsson L., Nielsen J. (2008). Metabolic footprinting in microbiology: Methods and applications in functional genomics and biotechnology. Trends Biotechnol..

[30] Chen W., Gong L., Guo Z., Wang W., Zhang H., Liu X., Yu S., Xiong L., Luo J. (2013). A Novel Integrated Method for Large-Scale Detection, Identification, and Quantification of Widely Targeted Metabolites: Application in the Study of Rice Metabolomics. Mol. Plant.

[31] Guo L., Milburn M.V., Ryals J.A., Lonergan S.C., Mitchell M.W., Wulff J.E., Alexander D.C., Evans A.M., Bridgewater B., Miller L. (2015). Plasma metabolomic profiles enhance precision medicine for volunteers of normal health. Proc. Natl. Acad. Sci. USA.

[32] Medina S., Dominguez-Perles R., Gil J., Ferreres F., Gil-Izquierdo A. (2014). Metabolomics and the Diagnosis of Human Diseases -A Guide to the Markers and Pathophysiological Pathways Affected. Curr. Med. Chem..

[33] Gibbons H., O’Gorman A., Brennan L. (2015). Metabolomics as a tool in nutritional research. Curr. Opin. Lipidol..

[34] Hall R.D. (2006). Plant metabolomics: From holistic hope, to hype, to hot topic. New Phytol..

[35] Uawisetwathana U., Karoonuthaisiri N. (2019). Metabolomics for rice quality and traceability: Feasibility and future aspects. Curr. Opin. Food Sci..

[36] Hu C., Xu G. (2013). Mass-spectrometry-based metabolomics analysis for foodomics. TrAC Trends Anal. Chem..

[37] Mozzi F., Ortiz M.E., Bleckwedel J., De Vuyst L., Pescuma M. (2013). Metabolomics as a tool for the comprehensive understanding of fermented and functional foods with lactic acid bacteria. Food Res. Int..

[38] Wishart D.S. (2019). NMR metabolomics: A look ahead. J. Magn. Reson..

[39] Larive C., Barding G.A., Dinges M. (2014). NMR Spectroscopy for Metabolomics and Metabolic Profiling. Anal. Chem..

[40] Ha D., Paulsen J., Sun N., Song Y.Q., Ham D. (2014). Scalable NMR spectroscopy with semico nductor chips. Proc. Natl. Acad. Sci. USA.

[41] Sundekilde U.K., Larsen L.B., Bertram H.C. (2013). NMR-Based Milk Metabolomics. Metabolites.

[42] Peterson A.L., Waterhouse A.L. (2016). 1H NMR: A novel approach to determining the thermodynamic properties of acetaldehyde con-densation reactions with glycerol, (+)-catechin, and glutathione in model wine. J. Agric. Food Chem..

[43] Noh M.F.M., Gunasegavan R.D.-N., Khalid N.M., Balasubramaniam V., Mustar S., Rashed A.A. (2020). Molecules Recent techniques in nutrient analysis for food composition database. Molecules.

[44] Singh D., Lee S., Lee C.H. (2017). Metabolomics for empirical delineation of the traditional Korean fermented foods and beverages. Trends Food Sci. Technol..

[45] Rocchetti G., O’Callaghan T.F. (2021). Application of metabolomics to assess milk quality and traceability. Curr. Opin. Food Sci..

[46] Trimigno A., Marincola F.C., Dellarosa N., Picone G., Laghi L. (2015). Definition of food quality by NMR-based foodomics. Curr. Opin. Food Sci..

[47] Rochfort S. (2005). Metabolomics Reviewed: A New “Omics” Platform Technology for Systems Biology and Implications for Natural Products Research. J. Nat. Prod..

[48] Yang Z. (2006). Online hyphenated liquid chromatography–nuclear magnetic resonance spectroscopy–mass spectrometry for drug metabolite and nature product analysis. J. Pharm. Biomed. Anal..

[49] Tabago M.K.A.G., Calingacion M.N., Garcia J. (2020). Recent advances in NMR-based metabolomics of alcoholic beverages. Food Chem. Mol. Sci..

[50] Li Y., Teng Z., Parkin K.L., Wang Q., Zhang Q., Luo W., Ma D., Zhao M. (2014). Identification of bioactive metabolites dihydrocana-densolide, kojic acid, and vanillic acid in soy sauce using GC-MS, NMR spectroscopy, and single-crystal x-ray diffraction. J. Agric. Food Chem..

[51] Lee S.H., Jung J.Y., Jeon C.O. (2015). Bacterial community dynamics and metabolite changes in myeolchi-aekjeot, a Korean traditional fermented fish sauce, during fermentation. Int. J. Food Microbiol..

[52] Naumann D., Helm D., Labischinski H. (1991). Microbiological characterizations by FT-IR spectroscopy. Nature.

[53] Kasprzyk I., Depciuch J., Grabek-Lejko D., Parlinska-Wojtan M. (2018). FTIR-ATR spectroscopy of pollen and honey as a tool for unifloral honey authentication. The case study of rape honey. Food Control..

[54] Wang F., Shao C., Chen Q., Meng T., Li C. (2019). Application on sensory prediction of Chinese moutai-flavor liquor based on ATR-FTIR. E3S Web Conf..

[55] Puxeu M., Andorra I., De Lamo-Castellví S., Ferrer-Gallego R. (2019). Determination of Nutrient Supplementation by Means of ATR-FTIR Spectroscopy during Wine Fermentation. Fermentation.

[56] Garcia-Hernandez C., Salvo-Comino C., Martin-Pedrosa F., Garcia-Cabezon C., Rodriguez-Mendez M. (2019). Analysis of red wines using an electronic tongue and infrared spectroscopy. Correlations with phenolic content and color parameters. LWT Food Sci. Technol..

[57] Krähmer A., Böttcher C., Gudi G., Stürtz M., Schulz H. (2021). Application of ATR-FTIR spectroscopy for profiling of non-structural carbohydrates in onion (*Allium cepa* L.) bulbs. Food Chem..

[58] Anjos O., Santos A.J.A., Estevinho L.M., Caldeira I. (2016). FTIR-ATR spectroscopy applied to quality control of grape-derived spirits. Food Chem..

[59] Ozulku G., Yildirim R.M., Toker O.S., Karasu S., Durak M.Z. (2017). Rapid detection of adulteration of cold pressed sesame oil adultered with hazelnut, canola, and sunflower oils using ATR-FTIR spectroscopy combined with chemometric. Food Control..

[60] Galvin-King P., Haughey S.A., Elliott C.T. (2020). Garlic adulteration detection using NIR and FTIR spectroscopy and chemometrics. J. Food Compos. Anal..

[61] Labaky P., Dahdouh L., Ricci J., Wisniewski C., Pallet D., Louka N., Grosmaire L. (2021). Impact of ripening on the physical properties of mango purees and application of simultaneous rheometry and in situ FTIR spectroscopy for rapid identification of biochemical and rheological changes. J. Food Eng..

[62] Kumar K., Giehl A., Patz C.-D. (2018). Chemometric assisted Fourier Transform Infrared (FTIR) Spectroscopic analysis of fruit wine samples: Optimizing the initialization and convergence criteria in the non-negative factor analysis algorithm for developing a robust classification model. Spectrochim. Acta Part A Mol. Biomol. Spectrosc..

[63] Li Z., Wang P.-P., Huang C.-C., Shang H., Pan S.-Y., Li X.-J. (2013). Application of Vis/NIR Spectroscopy for Chinese Liquor Discrimination. Food Anal. Methods.

[64] Wang L., Sun D.W., Pu H., Cheng J.H. (2017). Quality analysis, classification, and authentication of liquid foods by near-infrared spectroscopy: A review of recent research developments. Crit. Rev. Food Sci..

[65] Brunius C., Shi L., Landberg R. (2016). Large-scale untargeted LC-MS metabolomics data correction using between-batch feature alignment and cluster-based within-batch signal intensity drift correction. Metabolomics.

[66] Cajka T., Fiehn O. (2015). Toward Merging Untargeted and Targeted Methods in Mass Spectrometry-Based Metabolomics and Lipidomics. Anal. Chem..

[67] Allwood J.W., Goodacre R. (2010). An introduction to liquid chromatography-mass spectrometry instrumentation applied in plant metabolomic analyses. Phytochem. Anal..

[68] Mihailova A., Kelly S.D., Chevallier O.P., Elliott C.T., Maestroni B.M., Cannavan A. (2021). High-resolution mass spectrometry-based metabolomics for the discrimination between organic and conventional crops: A review. Trends Food Sci. Technol..

[69] Song H., Liu J. (2018). GC-O-MS technique and its applications in food flavor analysis. Food Res. Int..

[70] Dervishi E., Zhang G., Dunn S.M., Mandal R., Wishart D.S., Ametaj B.N. (2016). GC–MS Metabolomics Identifies Metabolite Alterations That Precede Subclinical Mastitis in the Blood of Transition Dairy Cows. J. Proteome Res..

[71] Fiehn O. (2016). Metabolomics by Gas Chromatography–Mass Spectrometry: Combined Targeted and Untargeted Profiling. Curr. Protoc. Mol. Biol..

[72] Moros G., Chatziioannou A.C., Gika H.G., Raikos N., Theodoridis G. (2017). Investigation of the derivatization conditions for GC–MS metabolomics of biological samples. Bioanalysis.

[73] Vinaixa M., Schymanski E., Neumann S., Navarro M., Salek R.M., Yanes O. (2016). Mass spectral databases for LC/MS- and GC/MS-based metabolomics: State of the field and future prospects. TrAC Trends Anal. Chem..

[74] Constantinou M., Louca-Christodoulou D., Agapiou A. (2021). Method validation for the determination of 314 pesticide residues using tandem MS systems (GC–MS/MS and LC-MS/MS) in raisins: Focus on risk exposure assessment and respective processing factors in real samples (a pilot survey). Food Chem..

[75] Li W., Chen Y.P., Blank I., Li F., Liu Y. (2021). GC×GC-TOF-MS and GC-IMS based volatile profile characterization of the chinese dry-cured hams from different regions. Food Res. Int..

[76] Magagna F., Cordero C., Cagliero C., Liberto E., Rubiolo P., Sgorbini B., Bicchi C. (2017). Black tea volatiles fingerprinting by compre-hensive two-dimensional gas chromatography-Mass spectrometry combined with high concentration capacity sample preparation techniques: Toward a fully automated sensomic assessment. Food Chem..

[77] Kuś P.M., Rola R. (2021). LC-QQQ-MS/MS methodology for determination of purine and pyrimidine derivatives in unifloral honeys and application of chemometrics for their classification. Food Chem..

[78] An F., Li M., Zhao Y., Zhang Y., Mu D., Hu X., You S., Wu J., Wu R. (2020). Metatranscriptome-based investigation of flavor-producing core microbiota in different fermentation stages of dajiang, a traditional fermented soybean paste of Northeast China. Food Chem..

[79] Lisanti M.T., Laboyrie J., Marchand-Marion S., de Revel G., Moio L., Riquier L., Franc C. (2021). Minty aroma compounds in red wine: Development of a novel automated HS-SPME-arrow and gas chromatography-tandem mass spectrometry quantification method. Food Chem..

[80] Ramautar R., Somsen G.W., De Jong G.J. (2014). CE-MS for metabolomics: Developments and applications in the period 2012–2014. Electrophoresis.

[81] Drouin N., Pezzatti J., Gagnebin Y., González-Ruiz V., Schappler J., Rudaz S. (2018). Effective mobility as a robust criterion for compound annotation and identification in metabolomics: Toward a mobility-based library. Anal. Chim. Acta.

[82] Yoshida M., Hatano N., Nishiumi S., Irino Y., Izumi Y., Takenawa T., Azuma T. (2011). Diagnosis of gastroenterological diseases by metabolome analysis using gas chromatography–mass spectrometry. J. Gastroenterol..

[83] Miggiels P., Wouters B., van Westen G.J., Dubbelman A.-C., Hankemeier T. (2018). Novel technologies for metabolomics: More for less. TrAC Trends Anal. Chem..

[84] Montona M.R.N., Soga T. (2008). Metabolome analysis by capillary electrophoresis-mass spectrometry. J. Chromatogr. A..

[85] Zhang W., Ramautar R. (2020). CE-MS for metabolomics: Developments and applications in the period 2018–2020. Electrophoresis.

[86] Wilson A.D. (2015). Advances in Electronic-Nose Technologies for the Detection of Volatile Biomarker Metabolites in the Human Breath. Metabolites.

[87] Rocchi R., Mascini M., Faberi A., Sergi M., Compagnone D., Di Martino V., Carradori S., Pittia P. (2019). Comparison of IRMS, GC-MS and E-Nose data for the discrimination of saffron samples with different origin, process and age. Food Control..

[88] Ghasemi-Varnamkhastia M., Apetreib C., Lozanoc J., Anyogu A. (2018). Potential use of electronic noses, electronic tongues and biosensors as multisensor systems for spoilage examination in foods. Trends. Food Sci. Technol..

[89] Tan J., Xu J. (2020). Applications of electronic nose (e-nose) and electronic tongue (e-tongue) in food quality-related properties determination: A review. Artif. Intell. Agric..

[90] Xu S., Zhou Z., Tian L., Lu H., Luo X., Lan Y. (2018). Study of the similarity and recognition between volatiles of brown rice plant hoppers and rice stem based on the electronic nose. Comput. Electron. Agric..

[91] Yang C., Ding W., Ma L., Jia R. (2015). Discrimination and characterization of different intensities of goaty flavor in goat milk by means of an electronic nose. J. Dairy Sci..

[92] Wasilewski T., Migon D., Gebicki J., Kamysz W. (2019). Critical review of electronic nose and tongue instruments prospects in pharma-ceutical analysis. Anal. Chim Acta..

[93] Mohd Ali M., Hashim N., Aziz S.A., Lasekan O. (2020). Principles and recent advances in electronic nose for quality inspection of agricultural and food products. Trends. Food Sci. Tech..

[94] Xu J., Liu K., Zhang C. (2021). Electronic nose for volatile organic compounds analysis in rice aging. Trends Food Sci. Technol..

[95] Jo D., Kim G.-R., Yeo S.-H., Jeong Y.-J., Noh B.S., Kwon J.-H. (2013). Analysis of aroma compounds of commercial cider vinegars with different acidities using SPME/GC-MS, electronic nose, and sensory evaluation. Food Sci. Biotechnol..

[96] Hong Y., Noh B.-S., Kim H.-Y. (2015). Discrimination of doenjang samples using a mass spectrometry-based electronic nose and human sensory preference testing. Food Sci. Biotechnol..

[97] Lalaleo L., Hidalgo D., Valle M., Calero-Cáceres W., Lamuela-Raventós R.M., Becerra-Martínez E. (2020). Differentiating, evaluating, and classifying three quinoa ecotypes by washing, cooking and germination treatments, using 1H NMR-based metabolomic approach. Food Chem..

[98] Peng C., Yao G., Sun Y., Guo S., Wang J., Mu X., Sun Z., Zhang H. (2021). Comparative effects of the single and binary probiotics of Lacticaseibacillus casei Zhang and Bifidobacterium lactis V9 on the growth and metabolomic profiles in yogurts. Food Res. Int..

[99] Raja G., Jung Y., Jung S.H., Kim T.-J. (2020). 1H-NMR-based metabolomics for cancer targeting and metabolic engineering—A review. Process. Biochem..

[100] Shi B., Ding H., Wang L., Wang C., Tian X., Fu Z., Zhang L., Han L. (2021). Investigation on the stability in plant metabolomics with a special focus on freeze-thaw cycles: LC–MS and NMR analysis to Cassiae Semen (*Cassia obtusifolia* L.) seeds as a case study. J. Pharm. Biomed. Anal..

[101] Luo Y., Gao F., Chang R., Zhang X., Zhong J., Wen J., Wu J., Zhou T. (2021). Metabolomics based comprehensive investigation of Gardeniae Fructus induced hepatotoxicity. Food Chem. Toxicol..

[102] Cardoso S., Afonso T., Maraschin M., Rocha M. (2019). WebSpecmine: A Website for Metabolomics Data Analysis and Mining. Metabolites.

[103] Gromski P.S., Muhamadali H., Ellis D., Xu Y., Correa E., Turner M., Goodacre R. (2015). A tutorial review: Metabolomics and partial least squares-discriminant analysis—A marriage of convenience or a shotgun wedding. Anal. Chim. Acta.

[104] Cha K.H., Lee E.H., Yoon H.S., Lee J.H., Kim J.Y., Kang K., Park J.-S., Jin J.B., Ko G., Pan C.-H. (2018). Effects of fermented milk treatment on microbial population and metabolomic outcomes in a three-stage semi-continuous culture system. Food Chem..

[105] Ma C., Li X., Zheng C., Zhou B., Xu C., Xia T. (2021). Comparison of characteristic components in tea-leaves fermented by Aspergillus pallidofulvus PT-3, *Aspergillus sesamicola* PT-4 and *Penicillium manginii* PT-5 using LC-MS metabolomics and HPLC analysis. Food Chem..

[106] Wang H., Zhang S., Sun Y., Dai Y. (2013). ACE-Inhibitory Peptide Isolated from Fermented Soybean Meal as Functional Food. Int. J. Food Eng..

[107] Tasdemir S.S., Sanlier N. (2020). An insight into the anticancer effects of fermented foods: A review. J. Funct. Foods.

[108] Dey T.B., Chakraborty S., Jain K.K., Sharma A., Kuhad R.C. (2016). Antioxidant phenolics and their microbial production by submerged and solid state fermentation process: A review. Trends Food Sci. Technol..

[109] Ferri M., Serrazanetti D.I., Tassoni A., Baldissarri M., Gianotti A. (2016). Improving the functional and sensorial profile of cereal-based fermented foods by selecting Lactobacillus plantarum strains via a metabolomics approach. Food Res. Int..

[110] Zhao Y., Wu C., Zhu Y., Zhou C., Xiong Z., Eweys A.S., Zhou H., Dong Y., Xiao X. (2021). Metabolomics strategy for revealing the components in fermented barley extracts with lactobacillus plantarum dy-1—Sciencedirect. Food Res. Int..

[111] Zhang H., Wang L., Tan Y., Wang H., Yang F., Chen L., Hao F., Lv X., Du H., Xu Y. (2020). Effect of Pichia on shaping the fermentation microbial community of sauce-flavor Baijiu. Int. J. Food Microbiol..

[112] Chen G.-M., Huang Z.-R., Wu L., Wu Q., Guo W.-L., Zhao W.-H., Liu B., Zhang W., Rao P.-F., Lv X.-C. (2021). Microbial diversity and flavor of Chinese rice wine (Huangjiu): An overview of current research and future prospects. Curr. Opin. Food Sci..

[113] Khattab A.R., Guirguis H.A., Tawfik S.M., Farag M.A. (2019). Cheese ripening: A review on modern technologies towards flavor en-hancement, process acceleration and improved quality assessment. Trends Food Sci. Tech..

[114] Zhou Z., Jian D., Gong M., Zhu S., Li G., Zhang S., Zhong F., Mao J. (2020). Characterization of the key aroma compounds in aged Zhenjiang aromatic vinegar by gas chromatography-olfactometry-mass spectrometry, quantitative measurements, aroma recombination and omission experiments. Food Res. Int..

[115] Wang J., Yuan C., Gao X., Kang Y., Huang M., Wu J., Liu Y., Zhang J., Li H., Zhang Y. (2020). Characterization of key aroma compounds in Huangjiu from northern China by sensory-directed flavor analysis. Food Res. Int..

[116] Yang L., Fan W., Xu Y. (2021). GC × GC-TOF/MS and UPLC-Q-TOF/MS based untargeted metabolomics coupled with physicochemical properties to reveal the characteristics of different type daqus for making soy sauce aroma and flavor type baijiu. LWT.

[117] Deng X., Huang G., Tu Q., Zhou H., Li Y., Shi H., Wu X., Ren H., Huang K., He X. (2021). Evolution analysis of flavor-active compounds during artificial fermentation of Pu-erh tea. Food Chem..

[118] Park M.K., Kim Y.S. (2019). Comparative metabolic expressions of fermented soybeans according to different microbial starters. Food Chem..

[119] Wang P., Ma X., Wang W., Xu D., Sun Y. (2019). Characterization of flavor fingerprinting of red sufu during fermentation and the com-parison of volatiles of typical products. Food Sci. Hum. Well..

[120] Leejeerajumnean A., Duckham S.C., Owens J.D., Ames J.M. (2001). Volatile compounds in Bacillus-fermented soybeans. J. Sci. Food Agric..

[121] Kimura K., Yokoyama S. (2018). Trends in the application of Bacillus in fermented foods. Curr. Opin. Biotech..

[122] Gao Y.X., Xu B., Fan H.R., Zhang M.R., Zhang L.J., Lu C., Na Zhang N., Fan B., Wang F.Z., Li S. (2020). 1H NMR-based chemometric metabolomics characterization of soymilk fermented by Bacillus subtilis BSNK-5. Food Res. Int..

[123] Wang Y., Shen Y., Wu Y., Li C., Li L., Zhao Y., Hu X., Wei Y., Huang H. (2021). Comparison of the microbial community and flavor compounds in fermented mandarin fish (*Siniperca chuatsi*): Three typical types of Chinese fermented mandarin fish products. Food Res. Int..

[124] Fang G.-Y., Chai L.-J., Zhong X.-Z., Jiang Y.-J. (2021). Deciphering the succession patterns of bacterial community and their correlations with environmental factors and flavor compounds during the fermentation of Zhejiang rosy vinegar. Int. J. Food Microbiol..

[125] Che H., Yu J., Sun J., Lu K., Xie W. (2021). Bacterial composition changes and volatile compounds during the fermentation of shrimp paste: Dynamic changes of microbial communities and flavor composition. Food Biosci..

[126] Thakur K., Tomar S.K., Wei Z.-J. (2017). Comparative mRNA Expression Profiles of Riboflavin Biosynthesis Genes in Lactobacilli Isolated from Human Feces and Fermented Bamboo Shoots. Front. Microbiol..

[127] Sharma S., Kandasamy S., Kavitake D., Shetty P.H. (2018). Probiotic characterization and antioxidant properties of Weissella confusa KR780676, isolated from an Indian fermented food. LWT.

[128] Thakur K., Tomar S.K., De S. (2015). Lactic acid bacteria as a cell factory for riboflavin production. Microb. Biotechnol..

[129] Zyalçin B., Sanlier N. (2020). The effect of diet components on cancer with epigenetic mechanisms. Trends Food Sci. Tech..

[130] Crespo L., Gaglio R., Martínez F.G., Martin G.M., Franciosi E., Madrid-Albarrán Y., Settanni L., Mozzi F., Pescum M. (2021). Bioaccumulation of selenium-by fruit origin lactic acid bacteria in tropical fermented fruit juices. LWT Food Sci. Technol..

[131] Daliri B.M., Tyagi A., Ofosu F.K., Chelliah R., Kim J.H., Kim J.R., Yoo D., Oh D.H. (2021). A discovery-based metabolomic approach using UHPLC Q-TOF MS/MS unveils a plethora of prospective antihypertensive compounds in Korean fermented soybeans. LWT Food Sci. Technol..

[132] Kang H.J., Yang H.J., Kim M.J., Han E.S., Kim H.J., Kwon D.Y. (2011). Metabolomic analysis of meju during fermentation by ultraper-formance liquid chromatography quadrupole-time of flight mass spectrometry (UPLC-Q-TOF MS). Food Chem..

[133] Sun X., Lyu G., Luan Y., Yang H., Zhao Z. (2019). Metabolomic study of the soybean pastes fermented by the single species *Penicillium glabrum* GQ1-3 and *Aspergillus oryzae* HGPA20. Food Chem..

[134] Namgung H.-J., Park H.-J., Cho I.H., Choi H.-K., Kwon D.-Y., Shim S.-M., Kim Y.-S. (2010). Metabolite profiling of doenjang, fermented soybean paste, during fermentation. J. Sci. Food Agric..

[135] Kim S.S., Kwak H.S., Kim M.J. (2020). The effect of various salinity levels on metabolomic profiles, antioxidant capacities and sensory attributes of doenjang, a fermented soybean paste. Food Chem..

[136] Jang H.-H., Noh H., Kim H.-W., Cho S.-Y., Kim H.-J., Lee S.-H., Lee S.-H., Gunter M.J., Ferrari P., Scalbert A. (2020). Metabolic tracking of isoflavones in soybean products and biosamples from healthy adults after fermented soybean consumption. Food Chem..

[137] Piras C., Marincola F.C., Savorani F., Engelsen S.B., Cosentino S., Viale S., Pisano M.B. (2013). A NMR metabolomics study of the ripening process of the Fiore Sardo cheese produced with autochthonous adjunct cultures. Food Chem..

[138] Bai M., Huang T., Guo S., Wang Y., Wang J., Kwok L.-Y., Dan T., Zhang H., Bilige M. (2020). Probiotic Lactobacillus casei Zhang improved the properties of stirred yogurt. Food Biosci..

[139] Yang S., Yan D., Zou Y., Mu D., Li X., Shi H., Luo X., Yang M., Yue X., Wu R. (2021). Fermentation temperature affects yogurt quality: A metabolomics study. Food Biosci..

[140] Wu S., Li N., Li S.-J., Bhandari B., Yang B.-L., Chen X.D., Mao Z.-H. (2009). Effects of Incubation Temperature, Starter Culture Level and Total Solids Content on the Rheological Properties of Yogurt. Int. J. Food Eng..

[141] Alaa A.E., Sally S., Samia E., Hany E. (2018). Developing functional yogurt rich in bioactive peptides and gamma-aminobutyric acid related to cardiovascular health. LWT Food Sci. Technol..

[142] Shi M., Mathai M.L., Xu G., McAinch A.J., Su X.Q. (2019). The effects of supplementation with blueberry, cyanidin-3-O-β-glucoside, yoghurt and its peptides on obesity and related comorbidities in a diet-induced obese mouse model. J. Funct. Foods..

[143] Tufariello M., Rizzuti A., Palombi L., Ragone R., Capozzi V., Gallo V., Mastrorilli P., Grieco F. (2021). Non-targeted metabolomic approach as a tool to evaluate the chemical profile of sparkling wines fermented with autochthonous yeast strains. Food Control..

[144] Weiskirchen S., Weiskirchen R. (2016). Resveratrol: How much wine do you have to drink to stay healthy?. Adv. Nutr..

[145] Benbouguerra N., Hornedo-Ortega R., Garcia F., El Khawand T., Saucier C., Richard T. (2021). Stilbenes in grape berries and wine and their potential role as anti-obesity agents: A review. Trends Food Sci. Technol..

[146] Pugajeva I., Pērkons I., Górnaś P. (2018). Identification and determination of stilbenes by Q-TOF in grape skins, seeds, juice and stems. J. Food Compos. Anal..

[147] Li R.-Y., Zheng X.-W., Zhang X., Yan Z., Wang X.-Y., Han B.-Z. (2018). Characterization of bacteria and yeasts isolated from traditional fermentation starter (Fen-Daqu) through a 1H NMR-based metabolomics approach. Food Microbiol..

[148] Ishihara S., Inaoka T., Nakamura T., Kimura K., Tomita S. (2018). Nuclear magnetic resonance- and gas chromatography/mass spectrometry-based metabolomic characterization of water-soluble and volatile compound profiles in cabbage vinegar. J. Biosci Bioeng..

[149] Tomita S., Watanabe J., Kuribayashi T., Tanaka S., Kawahara T. (2021). Metabolomic evaluation of different starter culture effects on water-soluble and volatile compound profiles in nozawana pickle fermentation. Food Chem. Mol. Sci..

[150] Drake M.A., Delahunty C.M. (2017). Chapter 20—Sensory character of cheese and its evaluation. Cheese.

[151] Seo H.S., Lee S., Singh D., Shin H.W., A Cho S., Lee C.H. (2018). Untargeted metabolite profiling for koji-fermentative bioprocess unravels the effects of varying substrate types and microbial inocula. Food Chem..

[152] Kasprowicz-Potocka M., Zaworska A., Gulewicz P., Nowak P., Frankiewicz A. (2017). The effect of fermentation of high alkaloid seeds of Lupinus angustifolius var. Karo by *Saccharomyces cerevisieae*, *Kluyveromyces lactis*, and *Candida utilis* on the chemical and microbial composition of products. J. Food Process. Preserv..

[153] Ding M., Liu W., Peng J., Liu X., Tang Y. (2018). Simultaneous determination of seven preservatives in food by dispersive liquid-liquid microextraction coupled with gas chromatography-mass spectrometry. Food Chem..

[154] Tungkijanansin N., Alahmad W., Nhujak T., Varanusupakul P. (2020). Simultaneous determination of benzoic acid, sorbic acid, and pro-pionic acid in fermented food by headspace solid-phase microextraction followed by GC-FID. Food Chem..

[155] Oyedeji A.B., Green E., Adebiyi J.A., Ogundele O.M., Gbashi S., Adefisoye M.A., Oyeyinka S.A., Adebo O.A. (2020). Metabolomic approaches for the determination of metabolites from pathogenic microorganisms: A review. Food Res. Int..

